# Bilateral Complete Oculomotor Palsy in Tubercular Meningitis

**DOI:** 10.7759/cureus.11001

**Published:** 2020-10-17

**Authors:** Saurabh Gaba, Monica Gupta, Amtoj Singh Lamba, Arshia Bhardwaj, Harsheel Gupta

**Affiliations:** 1 General Medicine, Government Medical College and Hospital, Chandigarh, IND

**Keywords:** tubercular meningitis, tuberculosis, oculomotor, bilateral, third nerve, palsy, ptosis

## Abstract

A 20-year-old female presented to the emergency department with fever, anorexia, headache, and neck stiffness for two weeks with recent onset of diplopia and ptosis. She was found to have bilateral symmetrical and complete oculomotor palsy. The diagnosis of tubercular meningitis (TBM) was established on magnetic resonance imaging of the brain and cerebrospinal fluid examination. The oculomotor palsy was attributed to tubercular exudates along the ventral surface of midbrain. Although cranial nerves palsies are common in TBM, such a pattern is rarely seen and has been reported only in the context of tuberculoma in midbrain. She was treated with anti-tubercular therapy for nine months, but there was only partial recovery of the oculomotor function.

## Introduction

The central nervous system (CNS) affliction in tuberculosis can occur in the form of encephalopathy, meningitis, tuberculoma, brain abscess, arachnoiditis, and spondylitis (Pott’s disease) [1]. The host risk factors include human immunodeficiency (HIV) virus infection, malnutrition, alcoholism, malignancy, and immunosuppression [2]. Tubercular meningitis (TBM) is the most catastrophic form of extrapulmonary tuberculosis, and it carries a high risk of mortality and neurological deficits in survivors [1]. It has classically been described to progress through three stages. The prodromal phase with non-specific clinical features of lethargy, anorexia, fever, and malaise is followed by the meningitis phase with features of meningeal irritation such as headache, neck stiffness, and photophobia. The final encephalitic stage is characterized by mental status changes, focal neurologic deficits, hydrocephalus, and vasculitic infarcts [1]. Timely diagnosis and management are vital for a favorable outcome. In this report, we are describing a distinctive clinical presentation in the form of a meningitic illness accompanied by isolated bilateral complete oculomotor (third cranial nerve) palsy. The cerebrospinal fluid (CSF) evaluation and magnetic resonance imaging (MRI) study revealed florid features of TBM.

## Case presentation

A 20-year-old girl presented to the emergency department with history of low-grade fever, anorexia, diffuse headache, and neck stiffness for two weeks. Over the preceding two days, she developed double vision, photophobia, and difficulty in opening her eyelids. She had no significant past medical or surgical history and lived in a single-room house with her parents and two younger brothers, one of whom had been treated for pulmonary tuberculosis (TB) a year earlier. There was no history of cough, hemoptysis, dyspnea, or seizures.

**Figure 1:**
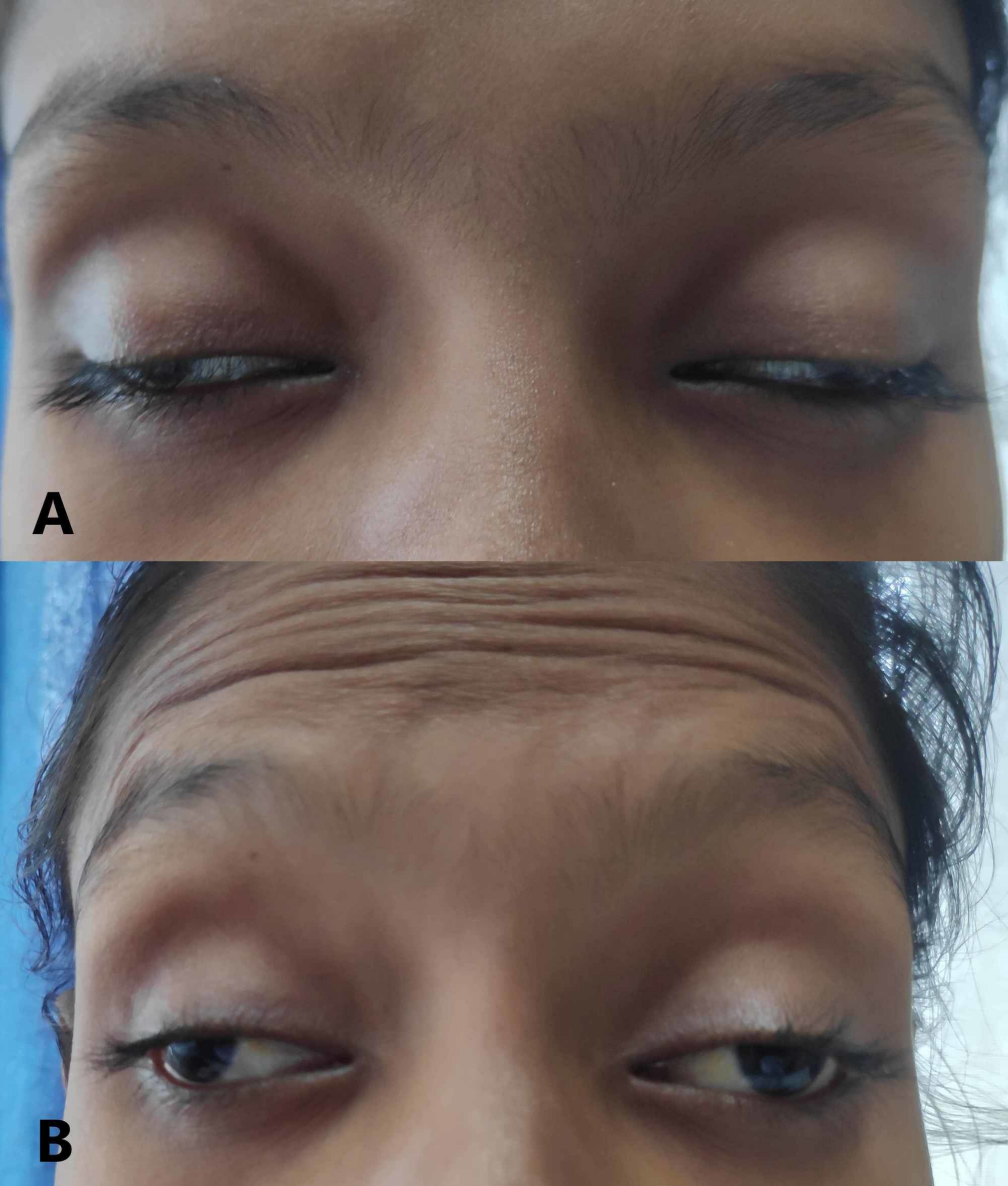
Bilateral complete oculomotor palsy. (A) The patient has ptosis, and eyes are deviated downward and laterally. (B) Eye opening is impaired due to paralysis of levator palpebrae superioris, so it requires contraction of the frontalis muscle, which causes wrinkling of the forehead.

On examination, her vitals were within normal limits, and she was conscious and oriented. She had no lymphadenopathy or enlargement of liver and spleen. Terminal neck rigidity was present, but Kernig's and Brudzinski's signs were negative. Visual acuity was normal, and she had a normal fundus without choroid tubercles or papilledema. There was complete ptosis, and eyes were deviated downward and laterally with dilated and non-reactive pupils, suggestive of bilateral complete oculomotor palsy (Figure 1). Examination of the other cranial nerves and rest of the nervous system did not reveal any abnormality.

**Figure 2:**
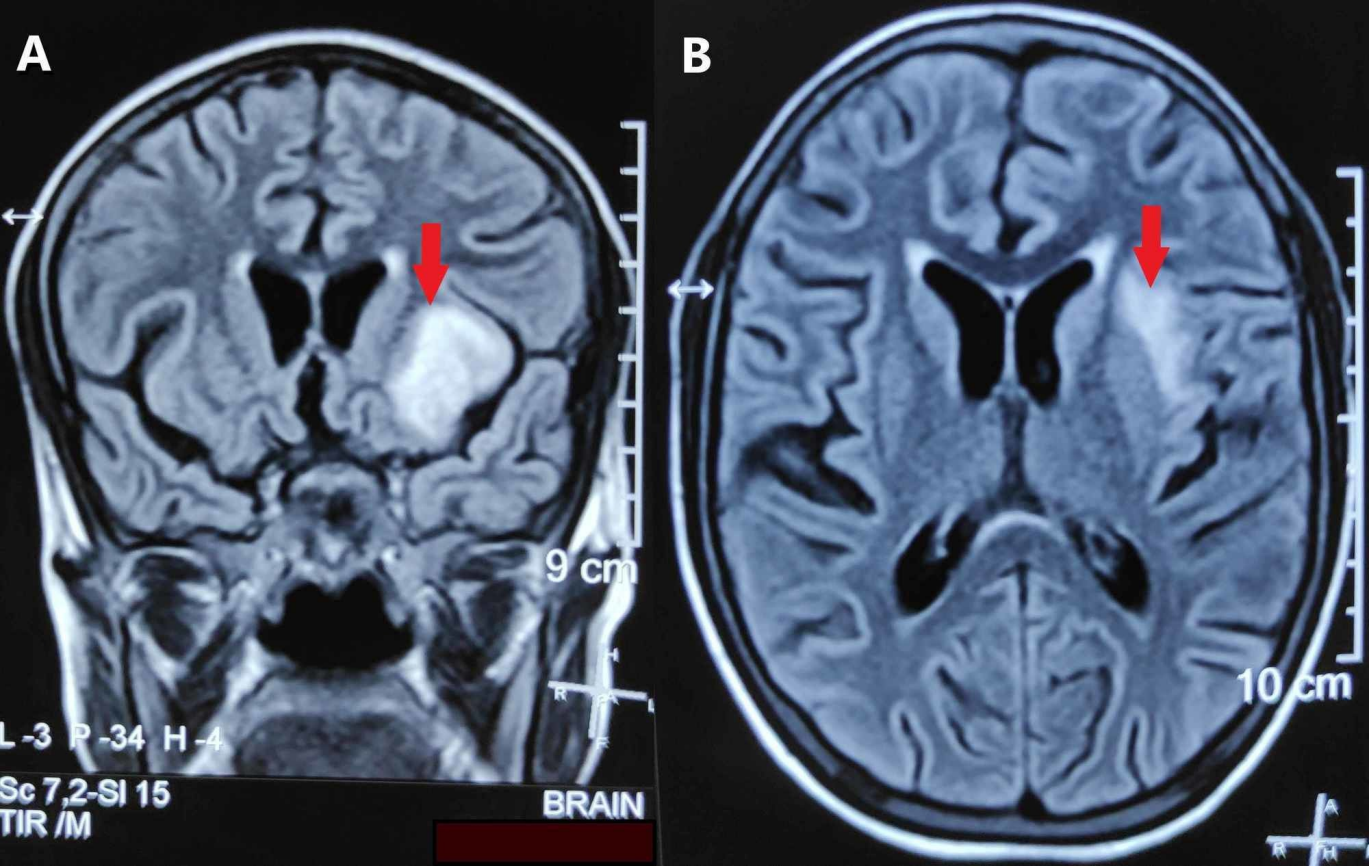
Coronal (A) and axial (B) sections of FLAIR sequence of MRI brain showing hyperintensity (red arrows) in left temporal lobe, suggestive of focal cerebritis. FLAIR, fluid-attenuated inversion recovery; MRI, magnetic resonance imaging.

**Figure 3:**
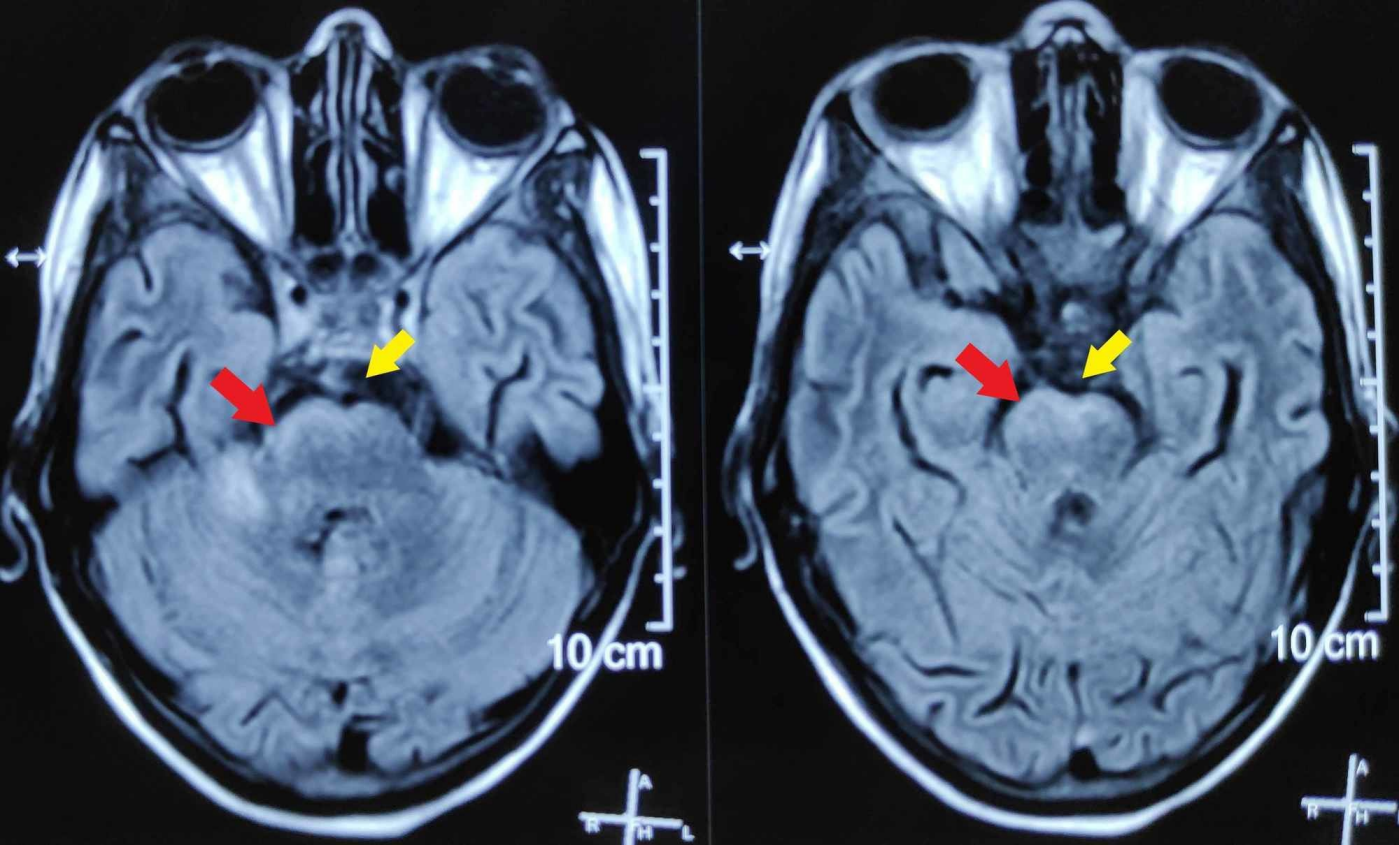
Axial sections of FLAIR sequence of MRI brain showing enhancement of the ventral surface of brainstem (red arrows) and basal exudates in the CSF (yellow arrows). FLAIR, fluid-attenuated inversion recovery; MRI, magnetic resonance imaging; CSF, cerebrospinal fluid.

**Figure 4:**
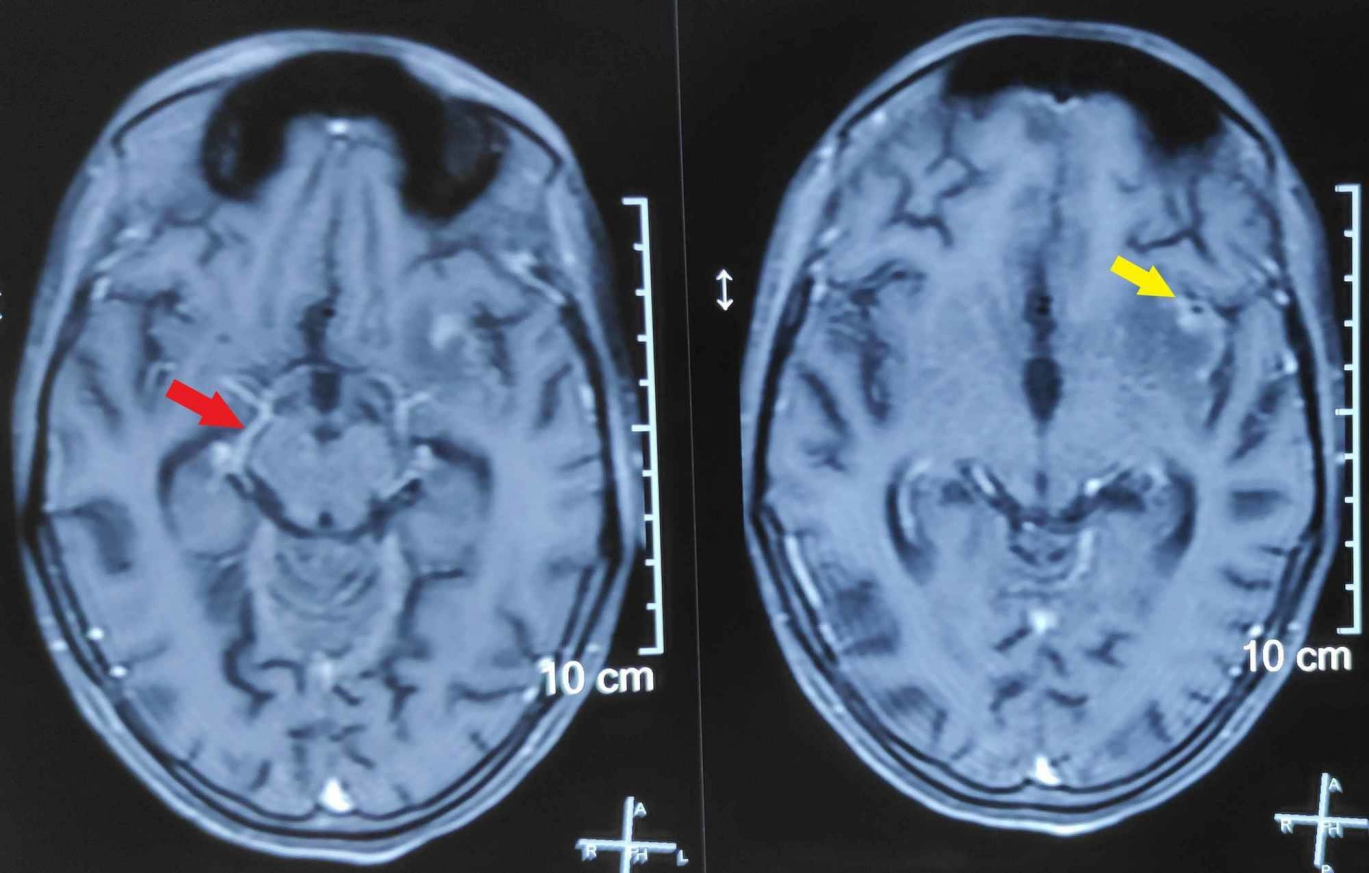
Axial sections of T1-GRE sequence of MRI brain showing basal (red arrow) and temporal (yellow arrow) meningeal enhancement. T1-GRE, T1-gradient recalled echo; MRI, magnetic resonance imaging.

**Figure 5:**
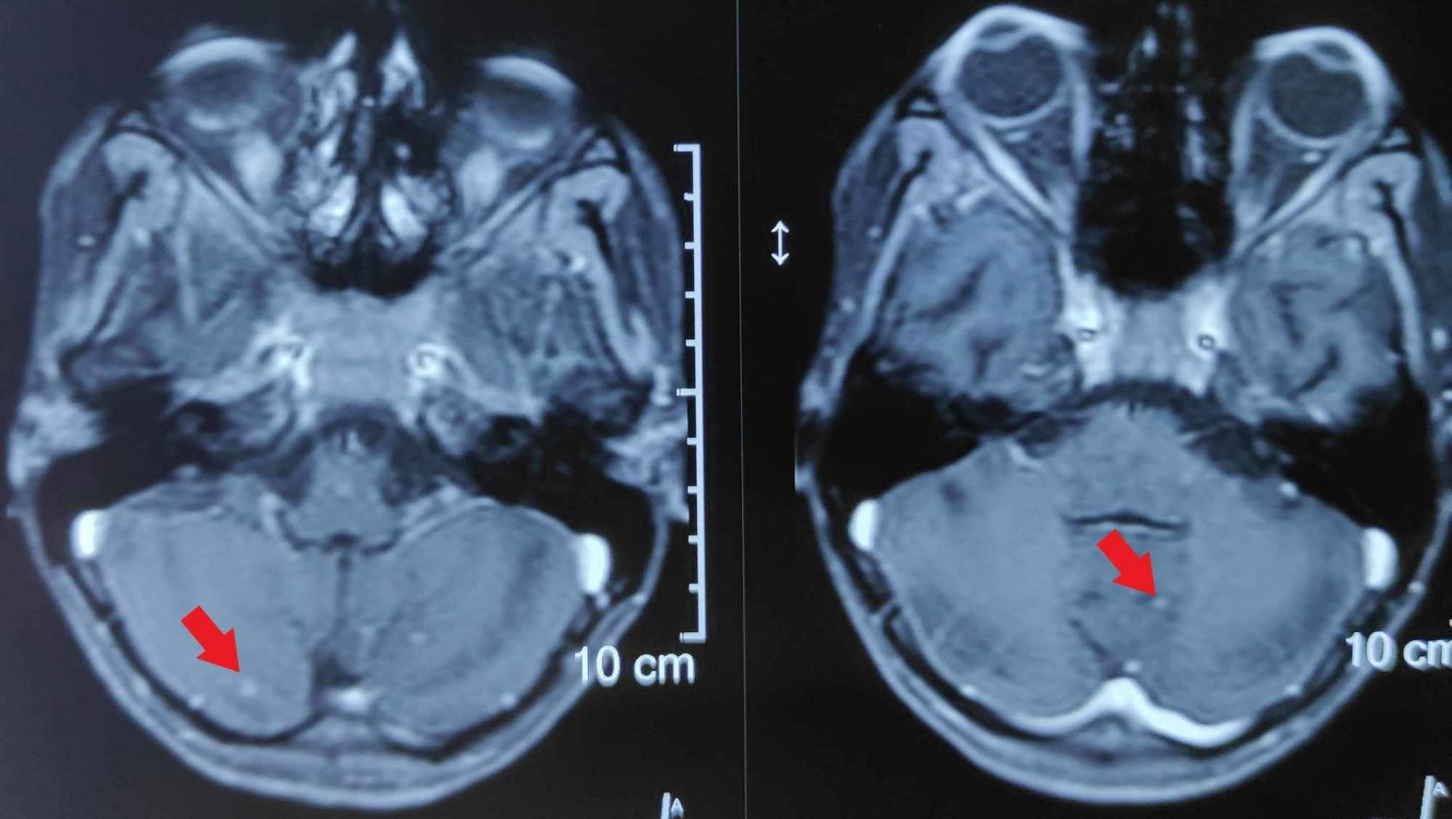
Axial sections of FLAIR sequence of MRI brain showing granulomas in the form of ring-enhancing lesions (red arrows) in cerebellum. FLAIR, fluid-attenuated inversion recovery; MRI, magnetic resonance imaging.

Investigations revealed hemoglobin of 10 gm/dL with microcytic hypochromic picture on the peripheral blood smear. Her erythrocyte sedimentation rate (ESR) was 75 mm in first hour. Her chest radiograph, ultrasound of the abdomen, liver function tests, and renal function tests were normal. She tested negative for HIV and antinuclear antibodies. The MRI of the brain (Figures 2-5) revealed features of focal temporal cerebritis with meningitis, cerebellar granulomas, and basal exudates. There was no vasculitic infarct, hydrocephalus, or tuberculoma. The CSF examination revealed an opening pressure of 25 cm of water, pleocytosis with total leucocyte count of 194/μL (70% lymphocytes and 30% neutrophils), protein of 160 mg/dL, glucose of 44 mg/dL, and adenosine deaminase (ADA) of 27.4 IU/L. The gram stain, acid fast staining, and culture did not reveal any organisms, but polymerase chain reaction (PCR) for *Mycobacterium tuberculosis* was positive with no rifampicin resistance detected. India ink staining and cryptococcal antigen assay were negative. The Mantoux test was positive with an induration of 15 mm.

**Figure 6:**
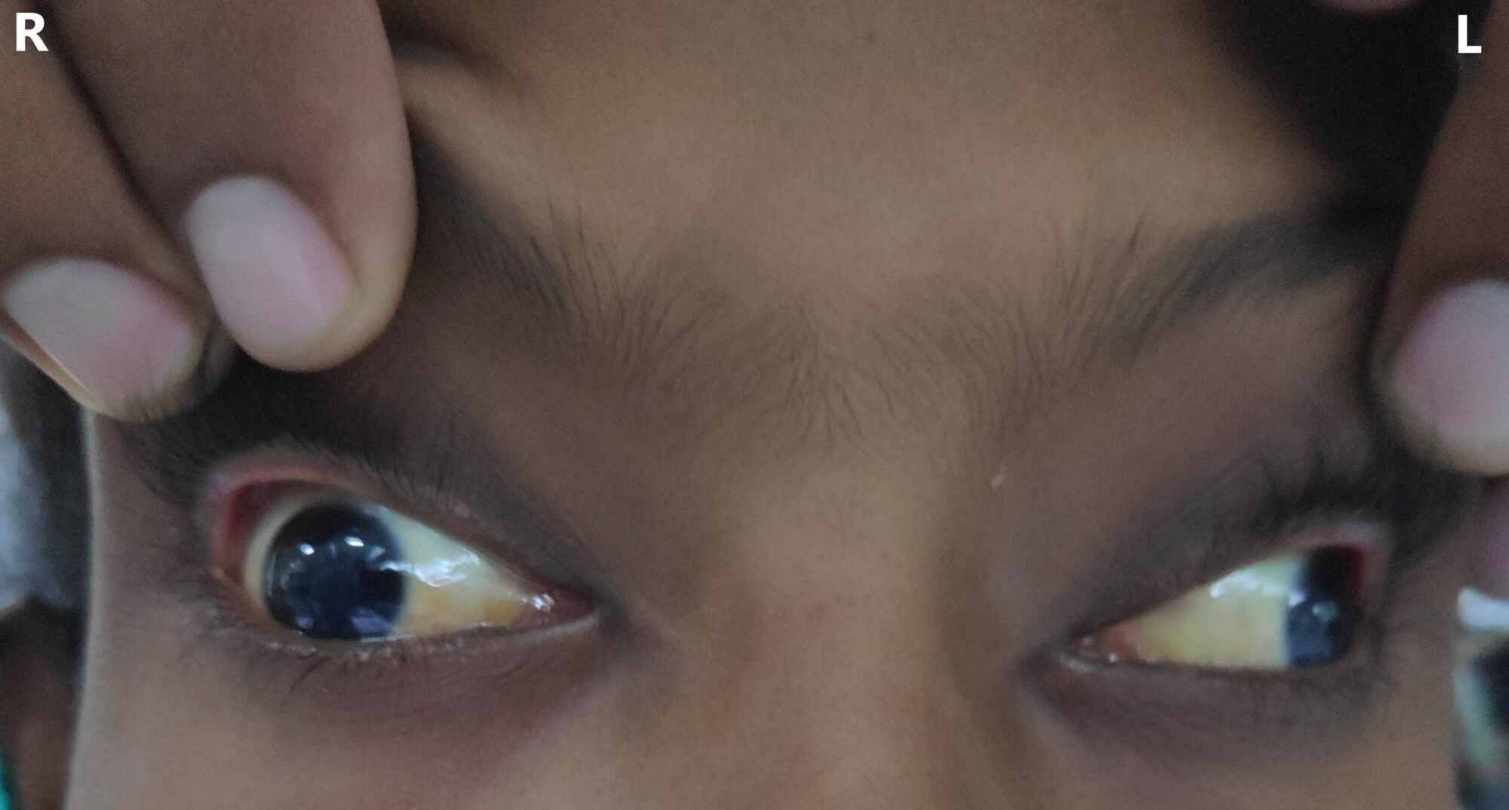
Partial recovery of the oculomotor function after treatment. In primary gaze position, the right eye (R) is slightly deviated while the left eye (L) has worsened with marked downward and lateral deviation.

She was treated with the standard anti-tubercular regime for nine months. Isoniazid, rifampicin, pyrazinamide, and ethambutol were given during the intensive phase for two months, followed by isoniazid and rifampicin for seven months. The treatment was supplemented with pyridoxine for the whole duration, and tapering dose of dexamethasone was given for the initial six weeks. There was incomplete recovery of the oculomotor function (Figure 6), and she used an eye patch to alleviate diplopia. A corrective ocular surgery was contemplated, but the patient refused it.

## Discussion

Hematogenous dissemination of inhalationally acquired tubercle bacilli from the lungs to CNS leads to formation of caseating foci (Rich foci) in the meninges and the brain [2]. Rupture of these foci into the subarachnoid space induces a robust inflammatory response that produces the clinical manifestations of TBM. A distinctive pathological feature of TBM is the inflammatory exudate. It is a purulent and gelatinous material produced by the host immune response [1]. It is best visualized on the T2 or fluid-attenuated inversion recovery (FLAIR) sequence of MRI of the brain as heterogeneously hyperintense signal within CSF. Exudates at the base of brain can surround the brainstem and lead to cranial nerve palsies. The most commonly affected are the sixth (abducens), third (oculomotor), fourth (trochlear), and 12th (hypoglossal) cranial nerves. Cranial nerve involvement can also occur due to mass effect from tuberculoma or abscess, due to brainstem infarction, or as false localizing sign due to increased intracranial tension [1]. The exudates can also impair the drainage of CSF from the basal cisterns, leading to obstructive hydrocephalus. Extension around the cerebral blood vessels can induce vasospam or vasculitis with consequent infarction.

The important causes of third nerve palsy are posterior communicating artery aneurysm, stroke, diabetes mellitus, vasculitis, tumors, meningitis, and trauma [3-7]. If the underlying pathology is microvascular ischemia as in diabetes and vasculitis, the pupillary function is generally not affected because the autonomic parasympathetic fibers that cause pupillary constriction are located on the periphery of the cross-section of the nerve. The motor nucleus of oculomotor nerve is situated in the midbrain, at the level of superior colliculus [8]. It emerges from the ventral surface of brainstem and traverses the cavernous sinus to enter orbit through the superior orbital fissure. Its motor fibers innervate levator palpebrae superioris and all the extraocular muscles except the superior oblique and lateral rectus. The parasympathetic fibers originate from Edinger-Westphal nucleus, and they innervate the ciliary ganglion from where they supply the sphincter pupillae via short ciliary nerves [8]. Oculomotor palsy can be complete (all functions impaired) or incomplete (some functions spared), depending on the extent of damage and the number of fibers involved.

Isolated bilateral oculomotor nerve palsy in TBM is very rare. Gaur et al. have reported bilateral partial and pupil-sparing oculomotor palsy in a 19-year-old male due to a tuberculoma in midbrain [9]. Sarkar et al. have reported a similar finding in an 11-year-old boy who also had ataxia [10]. In a retrospective study of 486 patients with TBM, 14.8% had cranial nerve palsies, either single or multiple [11]. Optic, oculomotor, abducens, and auditory nerve were the most commonly affected. After treatment, 97% of them recovered function without any obvious sequel [11]. Adjunctive treatment with corticosteroids helps in reducing the incidence of death and neurologic disability in TBM, including cranial nerve palsies [12]. This effect is mediated by reduction of intracranial tension, cerebral edema, and inflammation in the brain parenchyma, subarachnoid space, and blood vessels.

## Conclusions

This report illustrates a case of TBM presenting as a febrile illness with features of meningeal irritation and a previously unreported finding of bilateral complete oculomotor palsy due to basal exudates. Such affliction of cranial nerves has been reported only in the setting of tuberculoma in the brainstem. Cranial nerve palsies are common in TBM, and their pathogenesis is multifactorial. Timely diagnosis and treatment along with use of corticosteroids generally lead to good outcome. However, the patient under consideration had incomplete recovery of oculomotor function, and she was left with permanent disability. TBM should be suspected in all patients having cranial nerve palsies in the setting of a meningitis-like illness.
